# Supplementary material for: Limited effects of source population identity and number on seagrass transplant performance
Source: PeerJ. 2017 Feb 28;5:e2972. doi: 10.7717/peerj.2972 (PMC5333546; doi:10.7717/peerj.2972)
Supplement: Supplemental Information 1 [file peerj-05-2972-s001.docx]

**Supplemental Info**

**Table S1**. Microsatellite loci used for genotyping. Na = number of alleles found in the 91 samples genotyped in this study.

**Locus Accession number Reference PCR multiplex Na**

CT-12 AJ249303 Reusch 2000 A 2

CT-3 AJ009898 Hughes and Stachowicz 2004 A 10

GA-3 AJ009901 Reusch et al. 1999 A 6

CT-19 AJ249304 Reusch 2000 A 4

ZMC12075 AM408838 Oetjen and Reusch 2007 B 11

ZMC13053 AM408840 Oetjen and Reusch 2007 B 6

CL32Contig2 AM408831 Oetjen and Reusch 2007 B 4

GA-2 AJ009900 Reusch et al. 1999 C 6

CT-35 AJ249305 Reusch 2000 C 21

***Average*** 7.8

**Table S2**: Pairwise differentiation of source populations. Above the diagonal are F_st_ values estimated for all pairs; below the diagonal are the oceanic distances (km) between sites. All values are significant at *p* < 0.001

|  | Nahant | East Harbor | Pleasant Bay | West Yarmouth |
| --- | --- | --- | --- | --- |
| Nahant | -- | **0.095** | **0.065** | **0.177** |
| East Harbor | 106.7 | -- | **0.133** | **0.239** |
| Pleasant Bay | 145.5 | 47.8 | -- | **0.093** |
| West Yarmouth | 141.0 | 66.4 | 37.9 | -- |

**Table S3**. Results of the statistical analyses of source population identity and diversity on shoot density in transplants located in East Harbor.

|  |  | Source Population (*df* = 3) | |  | Diversity (*df* = 1) | |  |
| --- | --- | --- | --- | --- | --- | --- | --- |
|  | Month (post-transplantation) | *F* | *P* |  | *F* | *P* |  |
|  | March (4) | 10.94 | 0.0023 |  | 8.12 | 0.0292 |  |
|  | May (6) | 20.31 | 0.0002 |  | 0.11 | 0.7557 |  |
|  | June (7) | 0.81 | 0.5167 |  | 0.65 | 0.4511 |  |
|  | July (8) | 2.06 | 0.1696 |  | 2.03 | 0.2040 |  |
|  | August (9) | 2.87 | 0.0899 |  | 2.87 | 0.1412 |  |
|  | September (10) | 1.56 | 0.2604 |  | 6.30 | 0.0459 |  |
|  | October (11) | 0.79 | 0.5280 |  | 5.77 | 0.0530 |  |

**Figure S1.** Experimental design: Treatments were assigned to plots in a complete randomized block design, with each block containing the four unique single source treatments (East Harbor (EH); Nahant (N); Pleasant Bay (PB); West Yarmouth (WY)) and the four unique multiple source treatments (West Yarmouth, Pleasant Bay, East Harbor (WY,PB,EH); Pleasant Bay, Nahant, East Harbor (PB,N,EH); West Yarmouth, Pleasant Bay, Nahant (WY,PB,N); West Yarmouth, Nahant, East Harbort (WY,N,EH). There were four blocks at each site, resulting in 32 plots per site. The combination shown in this figure is for East Harbor.

**Figure S3**. Effect of source population and test site on the mean number of shoots per cross-hair six months (upper panel) and seven months (lower panel) after transplantation. Letters indicate significant differences among treatments at each sample period (Tukey’s HSD, *p* < 0.05, means ± SE).


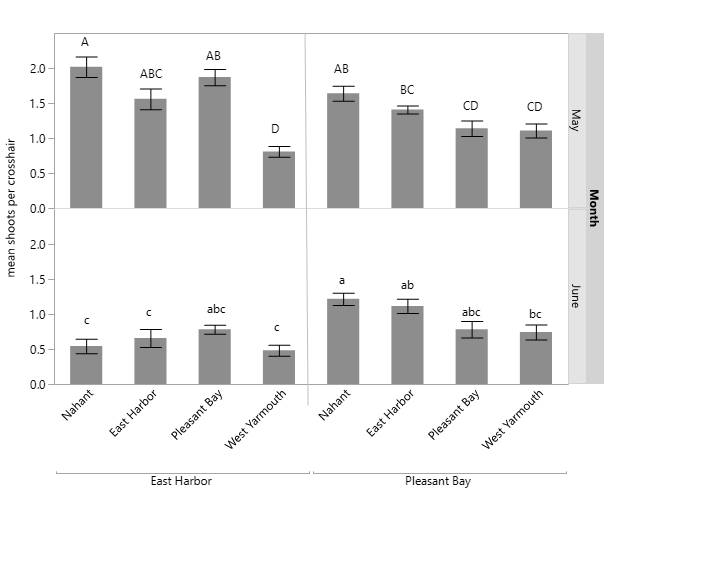


**Figure S4**. Effect of source diversity and transplant site on the mean number of shoots per cross-hair seven months after transplantation (June). The interaction between source diversity and site is marginally significant (*p* = 0.06, means ±SE).
